# Is maximum primary tumor diameter still a prognostic factor in patients with nasopharyngeal carcinoma treated using intensity-modulated radiotherapy?

**DOI:** 10.1186/s12885-015-1288-8

**Published:** 2015-04-18

**Authors:** Yong Chen, Xue-Feng Hu, Yan Wang, Hai-Yang Chen, Lin Yang, Li-Zhi Liu, Chun-Yan Cui, Dong-Sheng Liu, Shao-Bo Liang

**Affiliations:** 1 Department of Radiation Oncology, Sun Yat-sen University Cancer Center; State Key Laboratory of Oncology in South China; Collaborative Innovation Center for Cancer Medicine, Guangzhou, People’s Republic of China; 2 Radiotherapy Department of Head & Neck Carcinoma, Cancer Center, First People’s Hospital of Foshan Affiliated to Sun Yat-sen University, 81 Lingnan Street North, Foshan, People’s Republic of China; 3 Department of Radiation Oncology, the Sixth Affiliated Hospital of Sun Yat-sen University, Guangzhou, People’s Republic of China; 4 State Key Laboratory of Oncology in South China, Imaging Diagnosis and Interventional Center, Cancer Center, Sun Yat-sen University, Guangzhou, People’s Republic of China; 5 Department of Medical Statistics, First People’s Hospital of Foshan Affiliated to Sun Yat-sen University, Foshan, People’s Republic of China

**Keywords:** Nasopharyngeal carcinoma, Maximum primary tumor diameter, Intensity-modulated radiotherapy, Prognostic factor, TNM stage

## Abstract

**Background:**

Intensity-modulated radiation therapy (IMRT) has represented a technical milestone that has facilitated the clinical implementation. The purpose of this study was to evaluate the prognostic value of maximum primary tumor diameter (MPTD) in patients with nasopharyngeal carcinoma (NPC) treated using IMRT.

**Methods:**

Five-hundred and sixty-six patients with non-metastatic, histologically-confirmed NPC were retrospectively reviewed. MPTD was measured using magnetic resonance imaging (MRI). All patients were treated using IMRT; 87.5% (456/521) of patients with Stage T3-T4/N1-N3 disease also received cisplatin-based chemotherapy. Receiver operating characteristic (ROC) curves were used to identify the optimal MPTD cut-off point and examine the prognostic value of combining MPTD with the current T classification criteria.

**Results:**

Median follow-up for all patients was 36 months (range, 1–52 months). The 3-year overall survival (OS), failure-free survival (FFS), distant metastasis-free survival (DMFS), and local relapse-free survival (LRFS) rates for patients with a MPTD ≤41 vs. >41 mm were 96.1% vs. 85.4%, 93.7% vs. 74.7%, 96.1% vs. 79.7%, and 98.1% vs. 92.9%, respectively (all *P* < 0.05). In multivariate analysis, MPTD was an independent prognostic factor for OS, FFS, DMFS and LRFS in all patients (all *P* < 0.05). Among stage T3-T4 patients, the 3-year OS, FFS, DMFS, and LRFS rates for patients with a MPTD ≤41 vs. >41 mm were 96.9% vs. 84.5%, 95.4% vs. 73.5%, 96.1% vs. 79.2%, and 99.3% vs. 92.6%, respectively (all *P* < 0.05). In multivariate analysis, MPTD was also an independent prognostic factor for OS, FFS and DMFS in stage T3-T4 patients (all *P* < 0.05), and the difference in LRFS was almost statistically significant (*P* = 0.05). ROC curves verified that inclusion of MPTD improved the predictive value of the current T classification criteria (*P* < 0.001).

**Conclusions:**

MPTD was an independent prognostic factor in patients with NPC treated using IMRT, and significantly improved the prognostic value of the current T classification criteria for NPC.

## Background

Compared to other head and neck carcinomas, nasopharyngeal carcinoma (NPC) has a number of unique characteristics. Firstly, in contrast to its very low incidence in most other regions of the world, there is a high incidence of NPC in China and other countries in Southeast Asia [1]. Secondly, keratinizing squamous cell carcinoma is the major histological type in non-endemic areas; however, more than 95% of cases in the endemic areas are non-keratinizing carcinoma [2,3]. Thirdly, unlike other head and neck cancers, NPC is closely associated with Epstein-Barr viral infection [4,5]. Fourthly, radiotherapy (RT) is the first choice and main treatment method for non-metastatic NPC.

The introduction of intensity-modulated radiation therapy (IMRT) in the late 20th century represented a milestone in RT techniques. IMRT enables the tumor to receive higher dose of radiation, provides a more conformal dose distribution than two-dimensional RT (2-DRT) and significantly reduces the dose to surrounding normal anatomical structures. Lee et al. initially reported that the 4-year local progression free survival rate for NPC after IMRT was 97% (in 67 patients, 70% of whom had stage III-IV disease) [6]. Subsequently, other studies have confirmed that IMRT leads to excellent local control in NPC [7,8].

The seventh edition of the American Joint Committee on Cancer (AJCC) staging system and the Chinese 2008 staging system for NPC are widely used in clinical work [9,10]. The T classifications of both systems are based on tumor invasion of anatomical structures and cranial nerve paralysis, and do not include tumor size. Primary gross tumor volume (GTV-P) is an important prognostic factor in NPC [11,12]. However, assessment of GTV-P is so time-consuming that it violates the basic requirements for staging systems to be simple and practical.

Liang et al. provided the first demonstration that maximum primary tumor diameter (MPTD) was an important prognostic factor for 5-year overall survival (OS), failure-free survival (FFS), distant metastasis-free survival (DMFS) and local relapse-free survival (LRFS) in NPC [13]. However, the techniques employed in that study have been replaced with superior techniques. Firstly, the patients received 2-DRT or three-dimensional conformal RT (3-DCRT) instead of IMRT, which not only offers local control, but also reduces RT-related toxicities in patients with NPC [14,15]. Secondly, the addition of concurrent chemotherapy to radiotherapy has improved the survival outcome of patients with loco-regionally advanced NPC [16-18]; however, only 46.6% (131/281) of the patients with Stage III-IVB disease received concurrent chemoradiotherapy [13]. Furthermore, it remains unknown whether the addition of MPTD could improve the prognostic value of the T classification system for NPC.

On the basis of this premise, we initiated a retrospective study of a large cohort of patients to evaluate the prognostic value of MPTD in patients with NPC treated by IMRT and to determine whether the prognostic value of the current T classification system could be improved when combined with assessment of MPTD.

## Methods

### Study population

The Institutional Review Board of the Sun Yat-sen University Cancer Center approved the retrospective study. Written consent was waived, while oral consent was obtained via telephone and documented by telephone recording.

If the participants were at the age of 18 or over it, the oral consent was obtained from the participants. Otherwise, it would be obtained from their parents or guardians. Between November 2009 and December 2012, 566 consecutive patients with newly-diagnosed, histologically-proven, non-metastatic NPC who were treated at Sun Yat-sen University Cancer Center were included in this retrospective study. The cohort included 415 males and 151 females (male:female ratio, 2.7:1) with a median age of 46 years (range, 14–80 years). Histologically, 99.8% (565/566) of patients had non-keratinizing NPC; 0.2% (1/566) had keratinizing NPC. All patients underwent a pretreatment evaluation that included a complete medical history, physical and neurological examinations, hematology and biochemistry profiles, MRI scan of the nasopharynx and neck, chest radiography and abdominal sonography. Medical and imaging records were retrospectively reviewed, and all patients were restaged according to the 7th edition of the AJCC. The TNM stage distribution for all patients was 25.8% for T1, 17.5% for T2, 39.4% for T3, and 17.3% for T4; 16.1% for N0, 60.2% for N1, 20% for N2, and 3.7% for N3; 5.8% for stage I, 27.9% for stage II, 45.9% stage III, and 20.3% stage IVA-B.

### Imaging protocol

All patients underwent MRI using a 1.5-Tesla system (Signa CV/i; General Electric Healthcare, Chalfont St. Giles, United Kingdom). The area from the suprasellar cistern to the inferior margin of the sternal end of the clavicle was examined using a head-and-neck combined coil. T1-weighted fast spin-echo images in the axial, coronal and sagittal planes (repetition time, 500–600 ms; echo time, 10–20 ms; 22 cm field of view; 256 × 512 frequency matrix), and T2-weighted fast spin-echo MRI in the axial plane (repetition time, 4,000-6,000 ms; echo time, 95–110 ms; 22 cm field of view; 256 × 512 frequency matrix) were obtained before injection of contrast material. After intravenous injection of gadopentetate dimeglumine (0.1 mmol/kg body weight Gd-DTPA; Magnevist; Bayer-Schering, Berlin, Germany), spin-echo T1-weighted axial and sagittal sequences and spin-echo T1-weighted fat-suppressed coronal sequences were performed sequentially, using similar parameters to before injection. The section thickness was 3–4 mm with a 1 mm interslice gap for the sagittal plane, and 5 mm with a 1 mm interslice gap for the coronal and axial planes.

### Image assessment

Two radiologists qualified in diagnostic imaging in China with ≥10 years clinical experience focusing on head and neck carcinoma evaluated the MR images separately. Every two weeks, any disagreements were resolved by consensus. Tumors and soft tissue had intermediate signal intensity on pre-Gd-DTPA-T1 and T2-weighted images and enhanced intensity on post-Gd-DTPA T1-weighted images, with tumor replacing the normal anatomy of the structure. MPTD, defined as the maximum diameter of the continuous, uninterrupted tumor signal on post-Gd-DTPA T1-weighted images, was measured in the axial, coronal and sagittal planes; the largest value was recorded as the MPTD [13,19].

### Treatment

All patients were treated using IMRT; the protocol has previously been reported in detail [20,21]. The prescribed dose was 68–70 Gy to the planning target volume (PTV) of GTV-P, 60–66 Gy to the PTV of the nodal gross tumor volume (GTV-N), 60–62 Gy to the PTV of CTV-1 (i.e. high-risk regions) and 54–56 Gy to the PTV of CTV-2 (i.e. low-risk regions and neck nodal regions) over 30–31 fractions. RT was delivered over one fraction daily, 5 days per week.

Based on institutional treatment guidelines, concurrent chemotherapy was recommended for Stage T1-2N1M0 disease and concurrent chemotherapy +/− induction chemotherapy or adjuvant chemotherapy for Stage III-IVb disease. Overall, platinum-based chemotherapy was administered to 87.5% (456/521) of patients with Stage T1-2N1M0 or Stage III-IVb disease. In the event of documented relapse or persistent disease, salvage treatments including after-loading, surgery or chemotherapy were provided when appropriate.

### Follow up and statistical analysis

Patients were assessed every three months during the first two years, and every six months thereafter until censored (death, loss of follow-up or study termination). With regards to the measured indices, OS was measured from assignment to the date of death from any cause; FFS indicated the first failure at any site; and LRFS and DMFS were recorded as the first local or remote failure, respectively. Distant metastases were diagnosed based on clinical symptoms, physical examinations and imaging methods including X-ray, bone scan, MRI, CT and abdominal sonography. Locoregional recurrence was established by fiberoptic endoscopy, biopsy and MRI.

All analyses were performed using SPSS software version 13.0 (SPSS, Chicago, IL, USA). Actuarial rates were calculated by the Kaplan-Meier method and the differences were compared using the log-rank test. Multivariate analyses with the Cox proportional hazards model were used to test for significant independent prognostic factors using a backward elimination strategy. All patients were randomly allocated to a training set (*n* = 189) and a test set (*n* = 377). Receiver operating characteristic (ROC) curve analysis was used to evaluate different cut-off points for MPTD in the training set. Then, the test set and all patients were stratified according to the optimum cut-off point. The area under the ROC curve was used to assess the prognostic value of MPTD. The criterion for statistical significance was set at α = 0.05 and *P*-values were based on two-sided tests.

## Results

### Distribution of MPTD by T stage and survival rates

**Figure 1:**
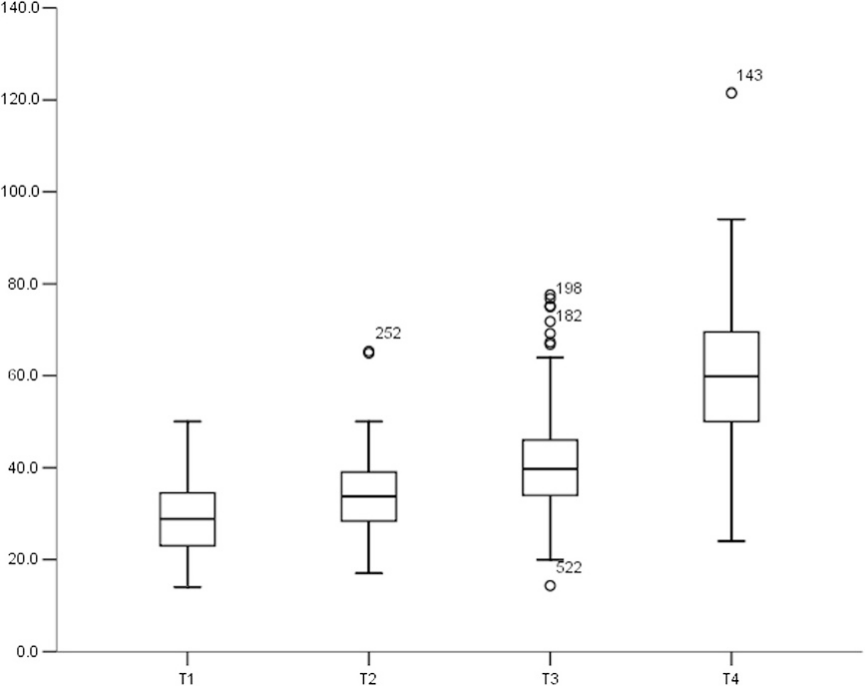
Distribution of maximum primary tumor diameter by T stage in 566 patients with NPC.

The distribution of MPTD by T stage is presented in Figure 1. The median MPTD was 28.7 mm (range, 14–50 mm) in T1, 33.3 mm (17–65.3 mm) in T2, 39.7 mm in T3 (14.3-77.6 mm), and 59.6 mm in T4 (24–121.5 mm). The MPTD values varied widely within the same T stage, and overlapped between different T stages.

The median follow-up for all patients was 36 months (range, 1 to 52 months). In total, 9% (51/566) of patients developed distant metastases, 5.3% (30/566) developed locoregional recurrence and 7.4% (42/566) died. The 3-year OS, DFS, DMFS and LRFS rates were 92.4%, 86.9%, 90.3% and 96.4%, respectively.

### Identification and prognostic verification of the optimal MPTD cut-off point

The optimal cut-off point for MPTD with respect to OS in the training set (*n* = 189) was 41 mm (sensitivity 81.8%, specificity 66.9%; AUC [area under the ROC] = 0.74, *P* = 0.007). Therefore, we selected a uniform cut-off point of 41 mm (>41 vs. ≤41 mm) to classify the test set and all patients into high and low MPTD groups for survival analysis.

In the test set (*n* = 377), the 3-year OS, FFS, and DMFS rates for patients with a MPTD ≤41 vs. >41 mm were 95.1% vs. 89.2%, 92.7% vs. 74.1%, and 95.5% vs. 77.9%, respectively (all *P* < 0.05). The 3-year LRFS rates for patients with a MPTD ≤41 vs. >41 mm were 98.1% vs. 95.1% (*P* = 0.191).

### Prognostic significance of MPTD in all patients

**Figure 2:**
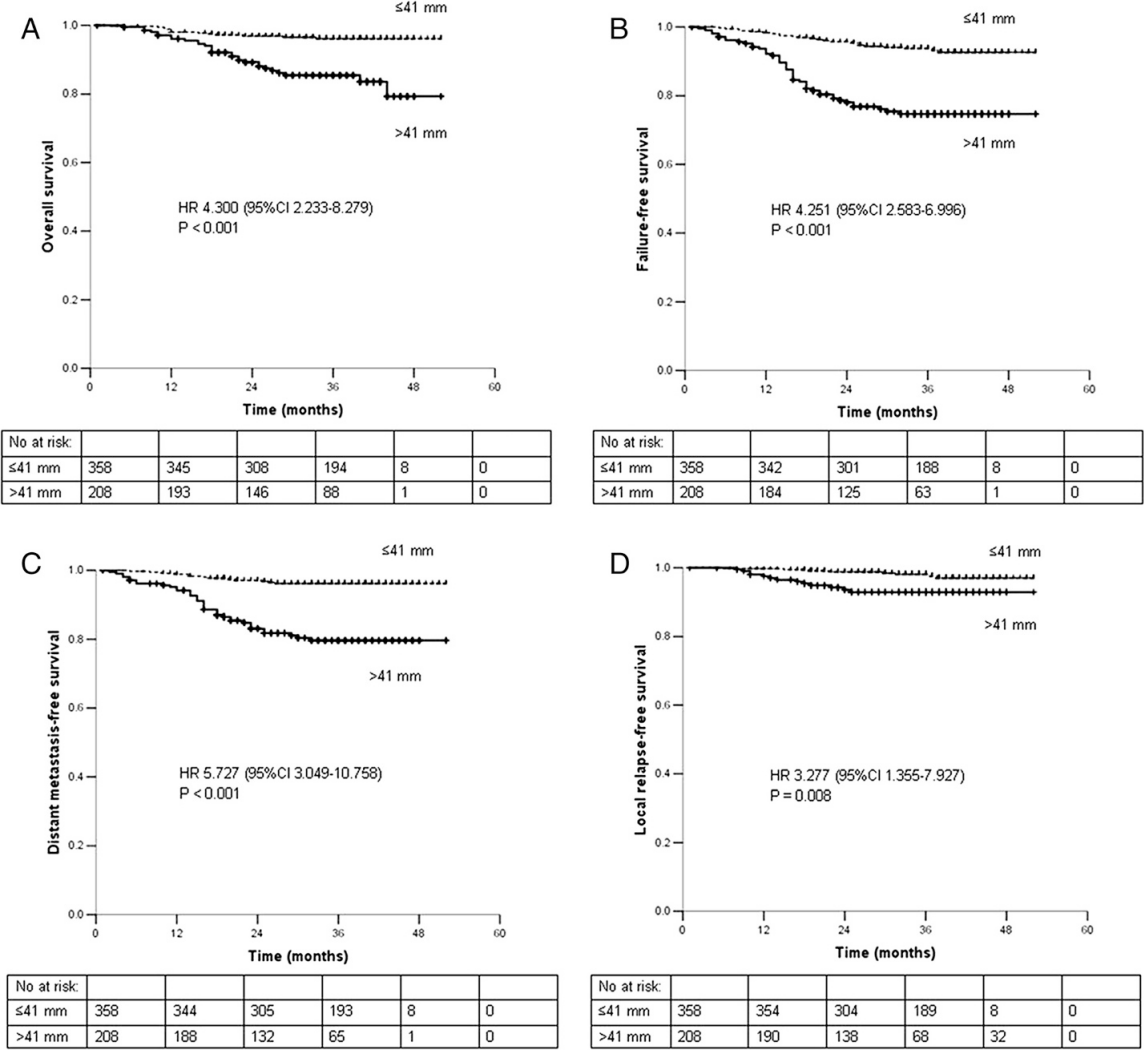
Survival rates of 566 patients with NPC stratified by maximum primary tumor Diameter. **A**. overall survival. **B**. failure-free survival. **C**. distant metastasis-free survival. **D**. local relapse-free survival. Abbreviations: 95% CI, 95% confidence interval; HR, hazard ratio; No at risk, number at risk.

In all patients (*n* = 566), the 3-year OS, FFS, DMFS, and LRFS rates for patients with a MPTD ≤41 vs. >41 mm were 96.1% vs. 85.4% (*P* < 0.001), 93.7% vs. 74.7% (*P* < 0.001), 96.1% vs. 79.7% (*P* < 0.001), and 98.1% vs. 92.9% (*P* = 0.008), respectively (Figure 2). The following parameters were included in the Cox proportional hazards model: age (≤45 vs. >45 years), sex, chemotherapy (yes vs. no) and additional boosts (yes vs. no), T stage (T1-2 vs. T3-4), N stage (N0-1 vs. N2-3) and MPTD (≤41 vs. >41 mm). MPTD was an independent prognostic factor for OS, FFS, DMFS and LRFS (all *P* < 0.05; Table 1).

**Table 1:** **Multivariate analyses of prognostic factors in 566 patients with nasopharyngeal carcinoma**

Endpoint	Variable	HR	95% CI	*P-*value
Overall survival	MPTD	4.300	2.233-8.279	<0.001
Failure-free survival	MPTD	3.968	2.402-6.559	<0.001
	N stage	1.802	1.111-2.923	0.017
Distant metastasis-free survival	MPTD	5.153	2.729-9.729	<0.001
	N stage	2.329	1.334-4.064	0.003
Local relapse-free survival	MPTD	3.277	1.355-7.927	0.008

*Abbreviations:* MPTD, maximum primary tumor diameter; 95% CI, 95% confidence interval; HR, hazard ratio.

### Prognostic significance of MPTD in patients with advanced T classification

**Figure 3:**
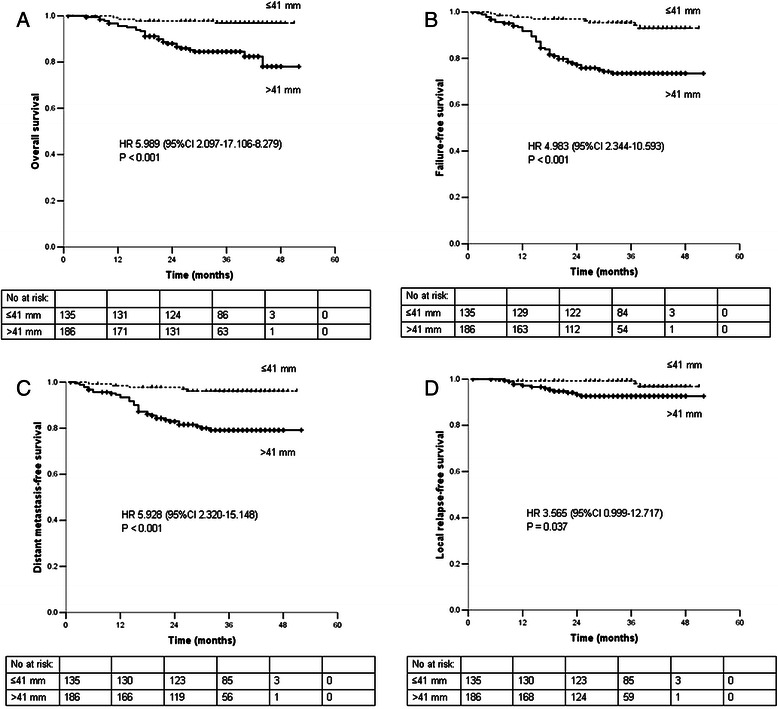
Survival rates of 321 patients with T3-T4 stage NPC stratified by maximum primary tumor diameter. **A**. overall survival. **B**. failure-free survival. **C**. distant metastasis-free survival. **D**. local relapse-free survival. Abbreviations: 95% CI, 95% confidence interval; HR, hazard ratio; No at risk, number at risk.

The 321 patients with T3-T4 stage disease were divided into two subgroups: Group 1 (T3-T4 disease with a MPTD ≤41 mm) and Group 2 (T3-T4 disease with a MPTD >41 mm). The 3-year OS, FFS, DMFS, and LRFS rates of the patients in Group 1 and Group 2 were 96.9% vs. 84.5% (*P* < 0.001), 95.4% vs. 73.5% (*P* < 0.001), 96.1% vs. 79.2% (*P* < 0.001), and 99.3% vs. 92.6% (*P* = 0.037; Figure 3). The following parameters were included in the Cox proportional hazards model: age (≤45 vs. >45 years), sex, chemotherapy (yes vs. no), additional boosts (yes vs. no), T stage (T3 vs. T4), N stage (N0-1 vs. N2-3) and MPTD (≤41 vs. >41 mm). MPTD was an independent prognostic factor for OS, FFS and DMFS (all *P* < 0.05), and the difference between LRFS was almost statistically significant (*P* = 0.05; Table 2).

**Table 2:** **Multivariate analyses of prognostic factors in 321 patients with nasopharyngeal carcinoma and advanced T classification**

Endpoint	Variable	HR	95% CI	*P-*value
Overall survival	MPTD	5.989	2.097-17.106	0.001
Failure-free survival	MPTD	4.592	2.149-9.816	<0.001
	N stage	1.797	1.031-3.131	0.039
Distant metastasis-free survival	MPTD	5.266	2.048-13.541	<0.001
	N stage	2.237	1.193-4.194	0.012
Local relapse-free survival	MPTD	3.565	0.999-12.717	0.050

*Abbreviations:* MPTD, maximum primary tumor diameter; 95% CI, 95% confidence interval; HR, hazard ratio.

### Predictive value of T classification combined with MPTD vs. T classification alone

**Figure 4:**
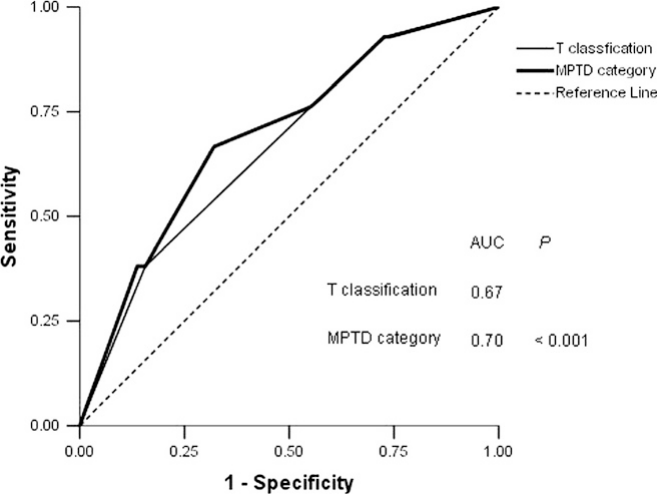
Receiver operator characteristic (ROC) curves for all patients with NPC (*n* = 566) when stratified by T classification combined with maximum primary tumor diameter (MPTD) and by T classification alone.

ROC curves were used to compare the predictive value of T classification combined with MPTD vs. T classification alone. In all patients, the AUC for T classification combined with MPTD (<41 and >41 mm) was 0.70, compared to 0.67 for T classification alone (*P* < 0.001; Figure 4). These results indicate that T classification combined with MPTD is superior to T classification alone for predicting prognosis.

## Discussion

IMRT has gradually replaced 2-DRT and 3-DCRT, and is currently the mainstream radiotherapy technique. As a result of the improved treatment outcomes provided in NPC by IMRT, it is necessary to reassess the prognostic factors identified by analyses of patients treated with 2-DRT and 3-DCRT [3,22,23]. This study demonstrates that MPTD was an independent prognostic factor for OS, FFS, DMFS and LRFS in patients with NPC treated using IMRT. Combining MPTD with the current criteria significantly improved the prognostic value of the T classification system for NPC.

### Prognostic value of MPTD in patients with NPC treated by IMRT

Larger MPTD values were more frequent in patients with higher T stage. However, the MPTD values varied widely within the same T stage, and overlapped between different T stages. These findings are in agreement with a previous study [13], and indicate that the current T classification does not accurately reflect tumor size in patients with NPC.

In the previous study by Liang et al., the patients were divided into three groups (≤30 vs. >30-50 vs. >50 mm) on the basis of the balance of the distribution of the MPTD values [13]. However, the ROC curve analysis was used to define the optimal cut-off points in this study. A MPTD cut-off point of 41 mm was selected for predicting OS. This cut-off point was validated in the test set, in which the 3-year OS, FFS and DMFS rates for patients with a MPTD ≤41 were significantly better than those of patients with a MPTD >41 mm (all *P* < 0.05).

MPTD (>41 vs. ≤41 mm) was an independent prognostic factor for OS, FFS, DMFS and LRFS in both univariate and multivariate analysis in all patients treated using IMRT. Liang et al. previously reported that MPTD (≤30 vs. >30-50 vs. >50 mm) was also an independent prognostic factor for OS, FFS, DMFS and LRFS in patients with NPC treated using 2-DRT or 3-DCRT [13]. Larger tumors may contain higher numbers of clonogenic tumor cells, possess larger areas of tumor hypoxia that promote resistance to radiotherapy and chemotherapy, or be associated with increased risk of distant micrometastases [24].

### Prognostic value of adding MPTD to the current T classification

In the stratified analysis of patients with stage T3-T4 disease, MPTD was an independent prognostic factor for OS, FFS and DMFS (all *P* < 0.05), and the difference in LRFS was nearly statistically significant (*P* = 0.05). Similar results were also observed in patients with NPC treated using 2-DRT or 3-DCRT [13]. This indicates that although advanced T classification disease is usually associated with poorer local control and shorter survival, patients within the same T classification with different MPTD values may have a different prognosis.

The current T classification does not include an assessment of tumor size, which this study demonstrates is an important prognostic factor in patients with NPC. Compared to the previous study by Liang et al., in addition to employing ROC curves analysis, we also investigated whether the prognostic value of the current T classification could be improved by adding MPTD. This study demonstrates that including MPTD in the current T classification enables superior prognostication compared to T classification alone (*P* < 0.001).

### Comparison of MPTD with GTV-P

In 1997, Chua et al. reported that GTV-P values varied widely within each T stage and represented an independent prognostic factor for local control in NPC, which appeared to be more predictive than Ho’s T stage classification [11]. Subsequently, a number of studies confirmed that GTV-P was an important prognostic factor in NPC [12,25,26]. Guo et al. reevaluated the prognostic value of GTV-P in 694 patients with NPC treated by IMRT, and confirmed that GTV-P was an independent prognostic factor that significantly improved the prognostic validity of T stage system [27].

MPTD and GTV-P, indexes reflecting the primary tumor size, are both important prognostic factors in patients with NPC. Compared to GTV-P, MPTD provides a less accurate assessment of tumor size; however, MPTD is quicker and easier to measure. Therefore, MPTD may be more convenient in clinical work and suitable for incorporation into the TNM staging system.

To the best of our knowledge, this is the first study to investigate the prognostic value of MPTD in patients with NPC treated by IMRT in a large number of patients. These results may help to refine the current T staging system for NPC. However, this was a retrospective study based on single-institution data, which needs to be confirmed by further multicentre studies.

## Conclusions

This study is the first attempt to evaluate the prognostic value of MPTD in patients with NPC treated by IMRT. Our analyses demonstrate that MPTD is also an independent prognostic factor for OS, FFS, DMFS and LRFS in patients with NPC treated by IMRT. Addition of MPTD may help to refine the prognostic value of the current staging system for NPC and assist with treatment strategy selection.
